# Detection of non-reference porcine endogenous retrovirus loci in the Vietnamese native pig genome

**DOI:** 10.1038/s41598-022-14654-4

**Published:** 2022-06-21

**Authors:** Shinya Ishihara, Masahiko Kumagai, Aisaku Arakawa, Masaaki Taniguchi, Ngo Thi Kim Cuc, Lan Doan Pham, Satoshi Mikawa, Kazuhiro Kikuchi

**Affiliations:** 1 Institute of Agrobiological Sciences, National Agriculture and Food Research Organization, Owashi 1-2, Tsukuba, Ibaraki 305-8634 Japan; 2 Advanced Analysis Center, National Agriculture and Food Research Organization, 2-1-2 Kannondai, Tsukuba, Ibaraki 305-8602 Japan; 3 Institute of Livestock and Grassland Science, National Agriculture and Food Research Organization, Ikenodai 2, Tsukuba, Ibaraki 305-0901 Japan; 4 National Institute of Animal Science, Hanoi, Vietnam; 5 Department of Animal Science, Nippon Veterinary and Life Science University, 1-7-1 Kyonancho, Musashino, Tokyo Japan 180-8602

**Keywords:** Animal breeding, Experimental models of disease, Interspersed repetitive sequences

## Abstract

The Vietnamese native pig (VnP)—a porcine breed with a small body—has proven suitable as a biomedical animal model. Here, we demonstrate that, compared to other breeds, VnPs have fewer copies of porcine endogenous retroviruses (PERVs), which pose a risk for xenotransplantation of pig organs to humans. More specifically, we sought to characterize non-reference PERVs (nrPERVs) that were previously unidentified in the reference genome. To this end, we used whole-genome sequencing data to identify nrPERV loci with long terminal repeat (LTR) sequences in VnPs. RetroSeq was used to estimate nrPERV loci based on the most current porcine reference genome (Sscrofa11.1). LTRs were detected using de novo sequencing read assembly near the loci containing the target site duplication sequences in the inferred regions. A total of 21 non-reference LTR loci were identified and separated into two subtypes based on phylogenetic analysis. Moreover, PERVs within the detected LTR loci were identified, the presence of which was confirmed using conventional PCR and Sanger sequencing. These novel loci represent previously unknown PERVs as they have not been identified in the porcine reference genome. Thus, our RetroSeq method accurately detects novel PERV loci, and can be applied for development of a useful biomedical model.

## Introduction

Northern Vietnam is a center of pig domestication^1^. Vietnamese native pigs (VnPs) have acquired unique biological characteristics through a long history of breeding and fixation^2^. We recently identified 32 populations of indigenous VnP breeds that widely differ in appearance^3^. Using single-nucleotide polymorphism array and microsatellite marker data^4,5^, the genetic characteristics of the VnP populations were found to be closely correlated with the geographic distribution of their habitats. However, certain VnP populations had been hybridized with exotic breeds, such as Landrace, imported for industrialized pig farming. Meanwhile, a recent study revealed that VnP genomes have lower porcine endogenous retrovirus (PERV) copy numbers than those of Western pig breeds^6^.

Endogenous retroviruses (ERVs) are viral elements integrated into the host genome. An exogenous retrovirus infection integrates the viral RNA genome as a provirus into the host genome. When this virus infects germline cells, the provirus is transmitted to the offspring as an ERV^7^. The ERV incorporated into the pig genome is known as a PERV and contains the functional genes *gag*, *pol*, and *env* with two long terminal repeats (LTRs) at the 5′ and 3′ ends of each locus. Typically, in the ERV, the functional genes *gag*, *pol*, and *env* encode proteins involved in viral particle formation, reverse transcriptase, as well as the glycoprotein of the viral envelope, which is associated with adhesion and invasion of host cells, respectively^8^.

Recombination occurs between the 5′ and 3′ LTRs to form solo-LTRs^9^. LTRs contain internal promoters and regulatory sequences, such as transcription factor binding sites, that alter the expression of adjacent host genes^9^. In fact, gene regulation by ERVs and solo-LTRs can alter the human phenotype^10,11^. Thus, determining the loci of PERVs, solo-LTRs corresponding to PERVs, and their neighboring functional genes is necessary to predict possible influences of PERVs on the host genome. In this way, the domestication and distinctive characteristics of VnPs may be better understood.

Moreover, PERVs are unfavorable genomic elements that can pose a significant risk for xenotransplantation of porcine tissues to human recipients. However, the copy number of PERV from VnPs, especially in the northern region of Vietnam was lower than that in other regions^6^. We collected pig samples from the Ban population in Yen Bai province (BanYB) to evaluate the PERV copy number. Depending on the PERV copies, this evaluation may help increase the gene modification success rate for producing PERV-free organisms. Moreover, due to their small body size, the organs of VnPs exhibit physiological similarities to those of humans^3^. As such, the VnP is expected to be representing a suitable biomedical model for producing xenotransplants for humans.

PERVs and solo-LTRs are dispersed in mutually similar sequences throughout the genome. It is, therefore, difficult to establish their precise genomic locations. Recently, whole-genome sequencing (WGS) data have enabled the examination of near-complete genomes for numerous species. To date, 20 PERVγ1 loci have been identified in swine using WGS. However, prior studies on PERV loci have been restricted to an earlier version (Sscrofa10.2) of the Duroc breed reference genome^12^. Therefore, non-reference PERVs (nrPERVs) may exist, however, do not appear in the reference genome. It is also possible that insertion loci (LTRs and PERV copy numbers) may vary among individuals and populations. In general, however, the reference genome was compiled without the repeat sequences (including ERVs) as integration of these elements has proven challenging. To address this issue, we have researched prior studies to identify a suitable method for detecting non-reference ERVs, as there is currently no established method for identifying nrPERVs in pigs.

The primary aim of the current study is to identify the nrPERV loci in VnPs to facilitate the establishment of a candidate biomedical model applicable for use in xenotransplantation. More specifically, we carried out quantitative real-time PCR (qRT-PCR) to analyze the PERV copy number as a simple measurement method. Collectively, we present the estimated numbers and loci of the LTRs and nrPERVs in the VnP genome.

## Results

### Sequencing data quality

**Figure 1:**
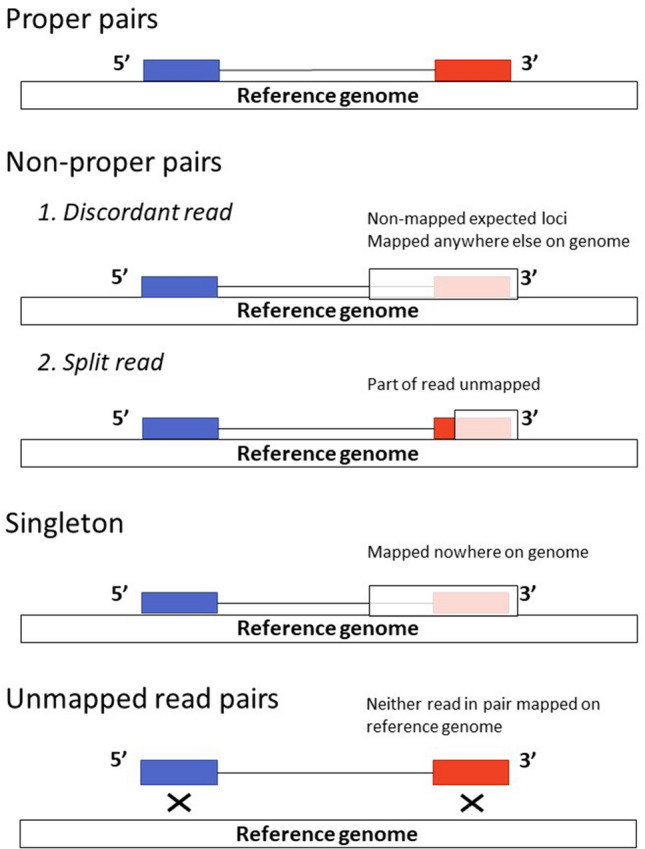
Conceptual diagram of sequencing reads mapping to the reference pig genome. White boxes denote an image of the pig reference genome sequence. Blue and red boxes connected with lines denote the 5′ and 3′ ends of a paired-end sequencing read. Most paired-end reads were identified as proper mapping while a small percentage of them were non-proper mapping. One end of the paired-end sequence mapped correctly while the other end was only partially identified at the expected locus on the reference genome. The unidentified sequence could be mapped anywhere else on the reference genome. For singletons, one end of the paired-end sequence mapped correctly while the other end did not map on the reference genome. For unmapped read pairs, neither read mapped to the reference genome.

We defined the three VnPs as VnP1, VnP2, and VnP3. qRT-PCR data indicated that the PERV *pol* gene copy numbers for VnP1, VnP2, and VnP3 were 7.3, 8.2, and 8.9, respectively. The PERV copy number identified with the RetroSeq method was then evaluated based on the qRT-PCR results. Data obtained by Illumina HiSeq X for these individuals are shown in Suppl. Table S2. The 150 bp paired-end reads exceeded, by more than 50-fold, the coverage of the entire genome for all three pigs. Trimming removed only 0.042% of the sequence reads and those remaining covered over forty-six-fold of the whole genome. The mapping results are shown in Suppl. Table S3. For each pig, > 94.2% of the paired-end reads mapped on the reference pig genome, whereas 1.63–1.69% did not. Moreover, 0.44–0.46% of the reads were singletons and were mapped on only one side. Non-proper pairs, such as discordant and split reads (Fig. 1), comprised 3.57–3.72% of the total genome. Sequence reads classified as non-proper pairs and singletons were used in the subsequent RetroSeq step. No quality control-failed reads were permitted to pass through the Burrows-Wheeler Alignment.

### In silico identification of the non-reference LTR breakpoint

**Figure 2:**
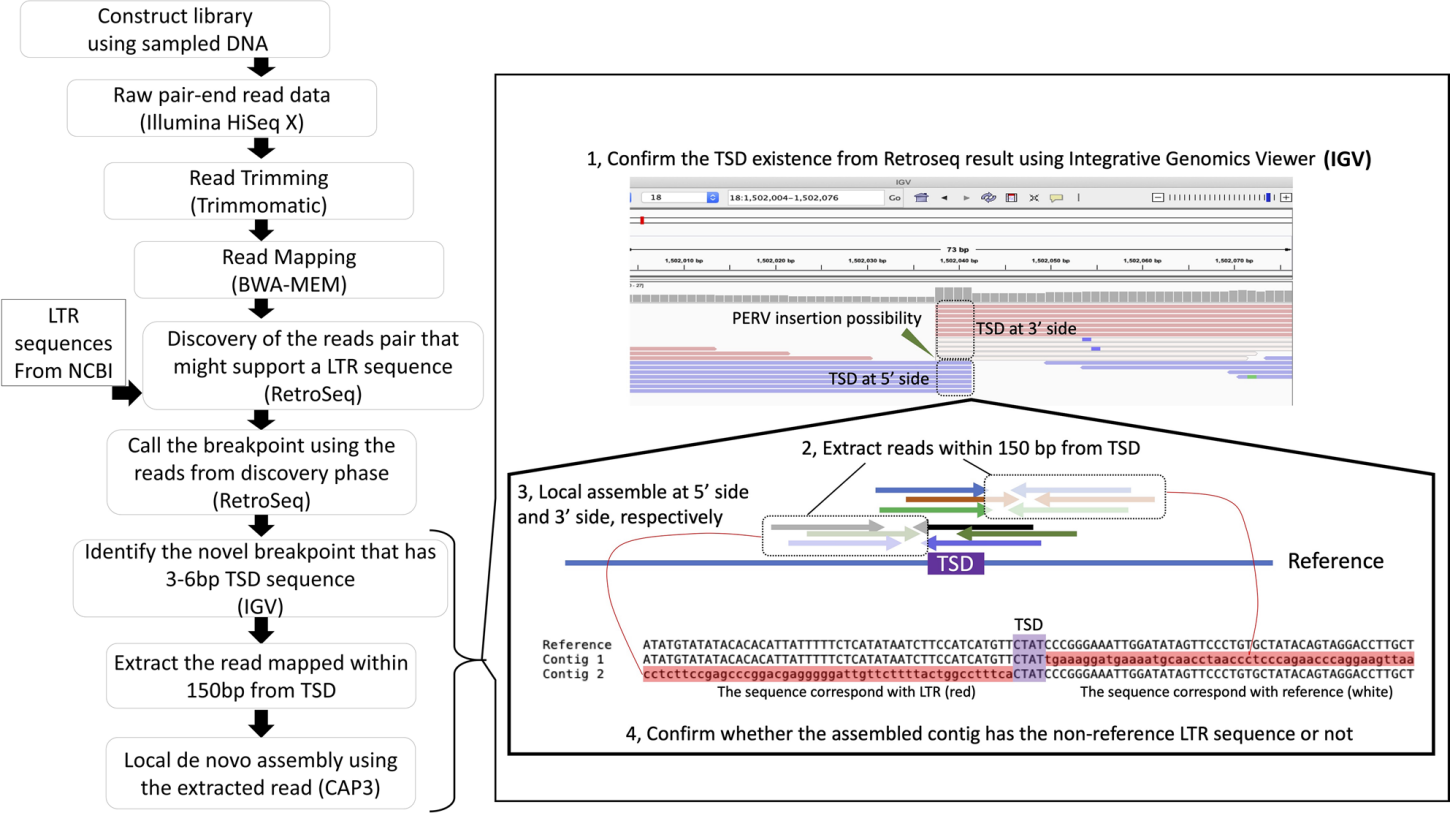
Pipeline for the detection of non-reference porcine endogenous retroviruses-long terminal repeats (PERV-LTRs) in whole-genome sequencing (WGS) read data. The presence of target site duplications (TSD) was confirmed at each locus detected by RetroSeq, extracted support reads from the TSD loci, performed local assembly, and analyzed the contigs for the presence of LTR-genome junctions from both sides. The upper panel (1) is a representative view of the integrative genomics viewer (IGV) used to determine potential PERV loci.

The analytical procedure is schematically represented in Fig. 2. In the RetroSeq “discover” step, we identified singleton and non-proper pairs among the read pairs supporting PERV-LTR (Fig. 2). We detected 8,884, 8,475, and 8,253 reads supporting LTRs in the genomic sequencing data of VnP1, VnP2, and VnP3, respectively. We then identified the LTR insertion loci (breakpoint) from the output of the RetroSeq “call” step. The candidate breakpoint was selected when the filter level was set to seven or eight, which is the range used in RetroSeq. A total of 220, 197, and 205 candidate LTR insertion loci were identified for VnP1, VnP2, and VnP3, respectively (Suppl. Table S4). We then used the merged Binary Alignment Map (BAM) data and Integrative Genomic Viewer (IGV) to detect 4–5 bp of target site duplication (TSD)-containing positions. TSDs were identified in loci on 23 autosomes and three X chromosomes. IGV mapping around the breakpoint showed that reads mapping on either the 5´ or 3´ end were broken at the TSD border (Fig. 2). Next, contigs were generated using a set of singleton and non-proper pair reads that mapped within 150 bp of the TSD and obtained them where one end matched the reference genome while the other did not (non-reference sequence). We then investigated whether these non-reference sequences matched the LTRs associated with PERV. Local de novo assembly generated the sequences containing the LTRs derived from all TSD-containing positions. The TSD sequences and the loci where the LTRs were detected in silico are shown in Table 1. The TSD sequences lacked any specific pattern. The lengths of the nrPERV-LTRs were in the range of 598–710 bp including their TSD sequences. The LTR sequences are shown in Suppl. Table S5. The contigs generated on the 5′ and 3′ ends of the TSD boundary were combined and the region between the TSDs was defined as the nrPERV-LTR sequence. Of the 26 LTRs, 21 were identified with TSDs at both the 5′ and 3′ ends. However, five LTRs were identified with only one TSD at either the 5′ or 3′ end. Therefore, we further analyzed the phylogenetics and LTR characteristics using 21 LTRs. The LTR chr13_57502585 harbored a mutation in the region overlapping combined contigs. Six other LTRs were identified within or around the functional genes (Table 1). Finally, PCR amplification and cycle sequencing analysis was performed on 26 LTR loci, from which nine, seven, and five nrPERV sequences were detected for VnP1, VnP2, and VnP3, respectively (Table 1).

**Table 1:** Detected target site duplication (TSD) position and sequences.

Chromosome	Position	TSD sequence	VnP1	VnP2	VnP3	Gene in the flanking region
**nrPERV confirmed with both 5′- and 3′-TSD**						
SSC 1	38,667,241	CTAT	LTR	LTR	LTR	*NKAIN2*
SSC 1	256,173,876	CCCC	PERV-B	PERV-B	LTR	ENSSSCG00000046664
SSC 1	259,647,577	AATC	LTR	LTR	LTR	N/A
SSC 2	3,400,723	AGAAC	PERV-B	LTR	LTR	N/A
SSC 4	77,324,504	CCCC	LTR	LTR	LTR	N/A
SSC 4	78,524,842	ATTAC	LTR	LTR	LTR	*SNTG1*^a^
SSC 4	121,221,912	GGGG	LTR	LTR	non-LTR	N/A
SSC 6	73,460,691	GTAT	LTR	LTR	LTR	*KAZN*
SSC 8	137,488,280	CTAT	LTR	LTR	LTR	*CFAP299*
SSC 9	61,533,579	GGTG	LTR	LTR	non-LTR	N/A
SSC 9	76,895,449	GAAC	PERV-B	PERV-B	PERV-B	N/A
SSC 9	135,717,008	AAGAG	LTR	LTR	LTR	N/A
SSC 12	60,076,460	CTGCT	PERV-B	PERV-B	PERV-B	LOC110256117
SSC 13	57,502,585	TAAA	LTR	LTR	LTR	N/A
SSC 13	60,210,737	GTAG	LTR	LTR	non-LTR	LOC106505659^c^
SSC 13	73,434,304	TTAT	LTR	LTR	non-LTR	N/A
SSC 14	4,896,607	AGGGT	LTR	LTR	non-LTR	N/A
SSC 14	27,599,572	ATGC	PERV-B	PERV-B	LTR	N/A
SSC X	70,665,683	ATAT	PERV-B	PERV-B	PERV-B	LOC102165634
SSC X	75,151,968	CCAG	PERV-B	PERV-B	PERV-B	*PCDH11X*
SSC X	119,479,008	AATT	LTR	LTR	non-LTR	N/A
**nrPERV confirmed with either 5′- or 3′-TSD**						
SSC 8	51,601,922	ATGA	PERV-C	PERV-C	PERV-C	LOC106504658^b^
SSC 8	137,628,915	ATGAC	non-LTR	non-LTR	LTR	*ANTXR2*
SSC 13	107,045,657	ATTC	PERV-A	non-LTR	non-LTR	LOC100153543
SSC 14	8,846,347	GAGG	LTR	LTR	non-LTR	N/A
SSC 18	4,030,456	ATGT	non-LTR	non-LTR	non-LTR	N/A

Chromosome number and position are based on the Sscrofa11.1 reference genome. Gene symbols are as follows: *NKAIN2*, Na + /K + transporting ATPase interacting 2; ENSSSCG00000046664, lncRNA; *SNTG1*, syntrophin gamma 1; *KAZN*, kazrin, periplakin interacting protein; *CFAP299*, cilia and flagella associated protein 299; *ANTXR2*, anthrax toxin receptor 2; LOC110256117; mRNA-multidrug and toxin extrusion protein 1-like, transcript variant; LOC100153543, multiple epidermal growth factor-like domains protein 10-like (predicted); *PCDH11X*, protocadherin 11 X-linked. LOC102165634, LOC106504658, and LOC106505659 are uncharacterized genes. N/A, not applicable.

^a^TSD position is 2.5 kb downstream of the gene.

^b^TSD position is 25 kb downstream of the gene.

^c^TSD position is 16 kb downstream of the gene.

### Non-reference PERV-LTR characteristics and phylogenetic analysis

**Figure 3:**
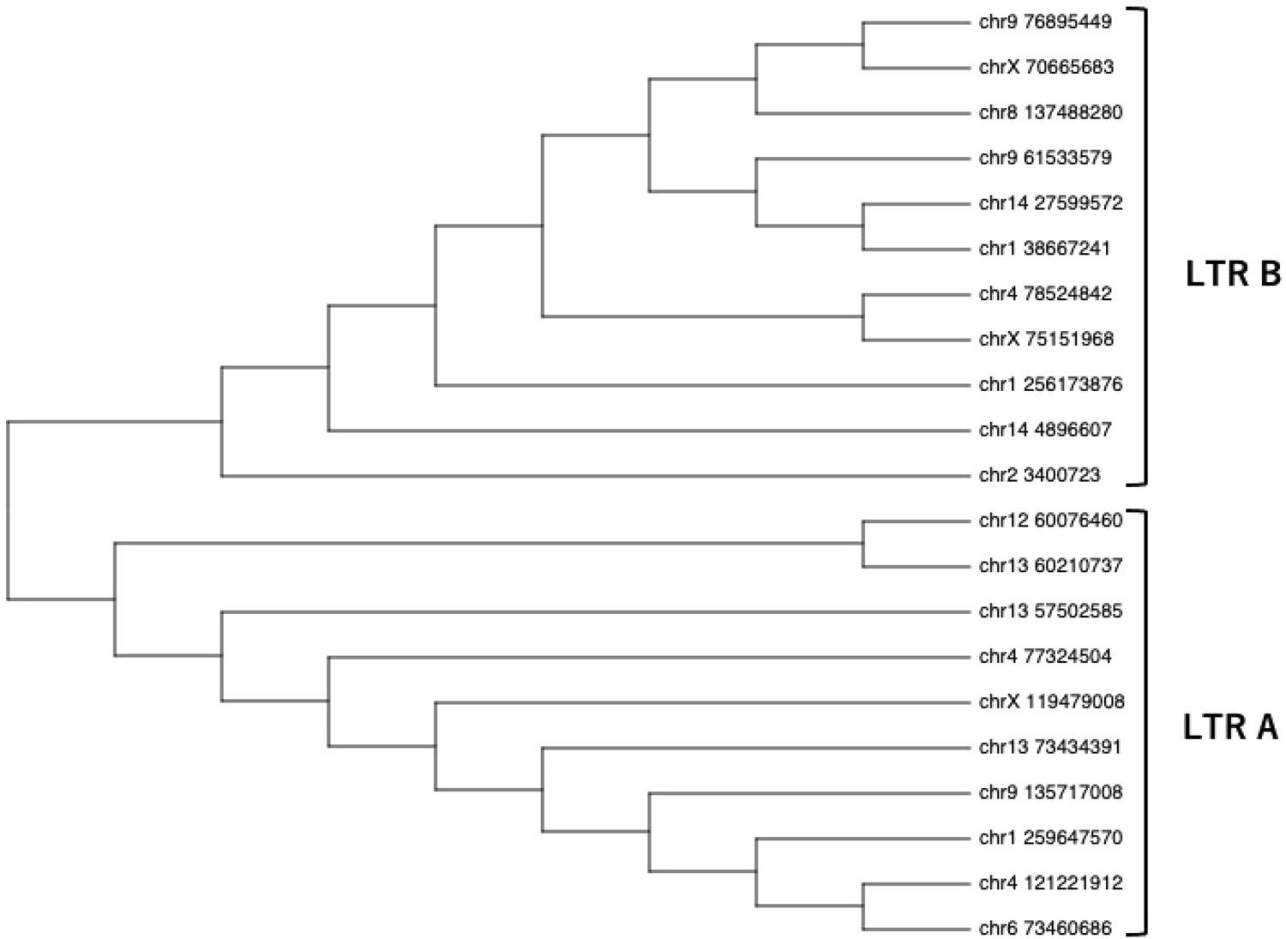
Phylogenetic tree of non-reference long terminal repeats (LTRs). The tree with the highest log likelihood (− 2693.75) is shown. A discrete gamma distribution was used to model the differences in evolutionary rate among sites (five categories; + G, parameter = 0.9509). This analysis involved 21 LTR sequences. There were 796 positions in the final dataset and two main clusters (LTR-A and LTR-B) were obtained.

**Figure 4:**
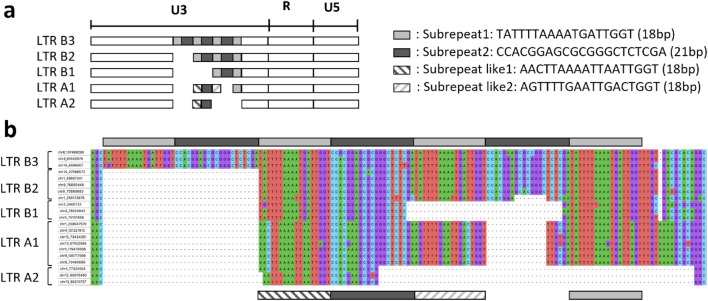
Structure for detecting non-reference long terminal repeats (LTRs) in the U3 region. (**a**) Porcine endogenous retroviruses (PERV)-LTR structure. The PERV-LTRs were classified into types B and A according to the patterns of their repeat sequences at 18 bp and 21 bp. Type B LTRs were divided into the subtypes LTR B1, LTR B2, and LTR B3 based on the number of repeats in their sequences. Type A LTRs were divided into the subtypes LTR A1 and LTR A2. (**b**) Type B repeat sequences are shown in light and dark gray at the top of the figure. Type A repeat sequences are shown in dark gray and stripes at the bottom of the figure. Nucleotides are denoted in green (A), blue (C), purple (G), and red (T). From top to bottom, the labels at left show the LTR loci chr8_137488280, chr9_61533579, chr14_4896607, chr14_27599572, chr1_38667241, chr9_76895449, chrX_70665683, chr1_256173876, chr2_3400723, chr4_78524842, chrX_75151968, chr1_259647570, chr4_121221912, chr13_73434391, chr13_57502585, chrX_119479008, chr9_135717008, chr6_73460686, chr4_77324504, chr12_60076460, and chr13_60210737.

Among the nrPERV-LTR sequences detected, there were several mutations, insertions, and deletions. In the maximum likelihood tree, the nrPERV-LTRs were classified into cluster LTR-A and LTR-B (Fig. 3). Of the 21 LTRs, 10 were classified as LTR-A and 11 as LTR-B. The LTR has U3, R, and U5 regions. We detected 18-bp and 21-bp repeats in the U3 region of LTR-B; however, the number of these repeats varied among LTR-B. In contrast, LTR-A lacked these repeats but had sub-repeat sequences resembling those of LTR-B (Fig. 4).

## Discussion

The sequencing data obtained here had a high depth even after trimming based on strict criteria. Over 94% of all read pairs mapped onto the reference pig genome. Hence, the assembled sequence data was deemed to be high in quality. Moreover, we attempted to detect nrPERVs with LTR (nrPERV-LTR) sequences using reads that were non-proper pairs and singleton sequences that did not map correctly to the reference genome. Hence, we detected 26 novel nrPERV-LTR loci in the porcine genome. Long PCR between the 5′-LTR and 3′-LTR confirmed the presence of PERVs and the existence of heretofore unreported nrPERV loci.

The RetroSeq-based method used in this study primarily targets non-reference LTR loci, which, in theory, were excluded from the reference pig genome. Previous studies identified PERV loci in the reference sequence of the pig genome using RetroTector software^13^. However, considering that these results did not overlap with those of the present study, the 26 nrPERV-LTR loci found herein are considered novel. Furthermore, RetroSeq analysis using WGS data and long PCR disclosed nine, seven, and five nrPERVs for VnP1, VnP2, and VnP3, respectively. Meanwhile, analysis of the whole-genome assembly (Sscrofa10.2) revealed 9 and 11 PERV-A and PERV-B loci, respectively^12^. However, we could not generate the corresponding data for these VnPs in the present study as the method used in this study is not applicable for detecting the PERV loci previously identified in the reference pig genome. Moreover, copy number values of 7.3, 8.2, and 8.9 were estimated for the PERVs in VnP1, VnP2, and VnP3, respectively, using qRT-PCR. As qRT-PCR could detect both the reference and non-reference LTRs, we could not to distinguish them. Thus, the qRT-PCR results may show lower values than the actual estimates. Overall, qRT-PCR is suitable for broad comparisons of PERV copy numbers among breeds; however, it cannot precisely discriminate them owing to bias effects resulting from PCR inhibitors and variable amplification efficiencies. In future, use of droplet digital PCR, which has been recognized as the most suitable method for absolute quantification of gene copy numbers, will help determine the PERV copy numbers in VnPs. This will enable comparison of results between the present and previous studies^14^.

The method used in the present study identified nrPERV loci and PERV types with greater accuracy than qRT-PCR as the former used long PCR validation. However, our methodology was restricted to non-reference genomes. It may be possible to improve nrPERV-LTR detection sensitivity and accuracy by increasing the amount of data or by adding long-read sequencing data. However, undetected PERVs at the “non-LTR” loci (Table 1) might exist. Certain loci might have been overlooked if long PCR amplification was inefficient when LTRs with repetitive sequences were present. In fact, our long PCR did not identify any LTR or PERV bands, even though LTR sequences were detected in silico for chr18_4030456. Although we designed primers from the candidate loci flanking sequences based on the Duroc reference genome, the VnPs used might have contained a mutation in the flanking sequence. Detection of all intact PERVs with LTRs required de novo assembly without the reference genome.

Seven of the nine nrPERV loci detected here were primarily PERV-B—the oldest phylogenetic PERV—while only one locus was found for PERV-A and PERV-C. These are all known competent subtypes of PERV which have high homology in the genes encoding *gag* and *pol*, however, differ in the genes encoding *env* proteins^15,16^. All members of the Suidae, including warthogs and red river hogs, harbor PERV-B. However, PERV-A and PERV-C are absent in warthogs while PERV-C is missing in red river hogs^17^. A study applying qRT-PCR reported that crossbreeding with Western species may increase PERV copy numbers^6^. Here, the copy numbers of PERV-A and PERV-C were lower than those of PERV-B. The latter commonly occurs in the wild boar and may have been conserved during the evolution of domesticated pigs. In contrast, the copy numbers of PERV-A and PERV-C are low in VnPs as this breed has occasionally been hybridized with Western species. Several studies have been conducted using different approaches including selection, short-interfering RNA, antibodies, and genome-editing technology (CRISPR/Cas9) to avoid PERV transmission during xenotransplantation^14,18–21^. The results of these previous studies suggest the possibility of producing PERV-free pigs, which can be used as breeding organisms to establish a novel biomedical model for xenotransplantation. For this purpose, additional genetic modification should be introduced into the VnP, because of their suitable size for humans and the presence of fewer PERV copies.

A phylogenetic tree was constructed using the LTR sequences detected in the current study. The sequences were divided into LTR-A and LTR-B (Fig. 3). LTR-A and LTR-B differ in terms of how many 18-bp and 21-bp repeats were present in the U3 region, and the presence or absence of alternating repeats (Fig. 4). The sequence characteristics determined here were consistent with those of previous reports^22–24^. The inserted LTRs act as host gene enhancers or promoters. In humans, the growth factor pleiotrophin has mitogenic, growth-promoting, and angiogenic properties and is expressed by the ERV-derived LTR promoter. The LTR promoter enables trophoblast-specific placental gene expression^25,26^. Regarding the porcine LTR, promoter activity increases when there are 39-bp repeats in the U3 region^22^. Some of the nrPERV-LTRs detected here were inserted along within functional genes. For example, the LTR detected in chr8_137488280 was LTR-B3 and was inserted into *CFAP299* with many repeats in the U3 region (Table 1; Fig. 4). Although *CFAP299* reportedly regulates murine spermatogenesis^27^, its precise function in pigs is unknown. Nevertheless, it is primarily expressed in the ovaries^28^ and, therefore, likely plays a role in reproduction. The LTRs detected in chr4_78524842 and chrX_75151968 were classified as LTR-B1. Each LTR was inserted into *SNTG1* and *PCDH11X*, respectively (Table 1). Though the functions of these genes in pigs remain unknown, *SNTG1* is associated with idiopathic scoliosis in humans^29^. Moreover, *PCDHX11* is related to the development of primary ovarian insufficiency in human females^30^ and might be involved in sexual maturation in cattle^31^. The relationships between certain LTRs and specific biological effects require further investigation. However, our observations suggest that the LTRs inserted within these genes might affect VnP traits.

LTRs have been implicated in evolutionary research. Studies have been conducted to estimate the time at which endogenous retroviruses were first inserted by comparing mutations between 5′ and 3′ LTRs^32^. Here, we detected a mutation between the 5′ and 3′ ends of the chr13_57502585 LTR. However, no mutations were detected in any of the other LTRs. Moreover, if chromosomal rearrangements occurred due to homologous recombination between distant proviruses, the flanking TSDs should differ. These points were mentioned in a previous study on ERV-mediated genome rearrangements in primates^33^. However, the TSDs detected in the present study were similar on both the 5′ and 3′ sides. Hence, the LTRs detected may have been recently inserted.

RetroSeq was used in the present study to identify novel PERV loci with LTRs not identified in the reference genome. The findings of this study contribute to the continued evaluation of whether pigs represent an ideal biomedical xenotransplantation model, however, PERV copy number is not the only factor to consider and further investigation is necessary in this regard.

## Methods

### Animal samples and genomic DNA purification

The animal experiments were conducted in compliance with the institutional rules for the Care and Use of Laboratory Animals and using a protocol approved by the Ministry of Agriculture and Rural Development, Vietnam (TCVN 8402:2010) and referred to the ARRIVE guidelines 2.0^34^. Blood samples were drawn from three sows collected from a pig farm only for breeding purposes in the Mu Can Chai District of Yen Bai Province, Vietnam^3^. No extra animal discomfort was caused for the blood sample collection for the purpose of this study. The population was defined as BanYB, as previously reported^3^. Genomic DNA was extracted from the blood samples with the QIAamp DNA Blood and Tissue Kit (Qiagen, Hilden, Germany). The DNA was then quantified with a Qubit dsDNA HS Assay Kit and a Qubit 2.0 fluorometer (Thermo Fisher Scientific, Waltham, MA, USA). DNA quality was evaluated by gel electrophoresis.

### Whole-genome sequencing

One milligram of DNA and a TruSeq DNA PCR-Free Sample Prep Kit (Illumina Inc., San Diego, CA, USA) were used to construct each 350-bp sequencing library. WGS was performed on 150-bp paired-end reads using an Illumina HiSeq X Platform (Illumina Inc.). Nucleotides in these reads with low-quality scores were trimmed and adapters were removed with Trimmomatic v. 0.36^35^ using the settings ILLUMINACLIP: TruSeq3-PE:2:30:10, LEADING: 3, SLIDINGWINDOW: 4:20, and MINLEN: 30. Reads were mapped to the *Sus scrofa* genome Build 11.1 (Sscrofa11.1; GCA_000003025.6) using a Burrows-Wheeler Aligner with a ‘mem’ algorithm^36^. The data were generated in BAM format. Raw WGS data were deposited in the DDBJ Sequence Read Archive under Accession No. DRA013149.

### Detection of non-reference LTRs

The types of read pairs that mapped to the reference genome were defined to extract sequencing reads that were useful for this research. Most paired end reads derived from WGS, map to the reference genome. However, discordant read pairs may also occur and may have unexpected span sizes/inconsistent orientations. The designation “non-proper pairs” refers to a 5′ or 3′ end that maps to a contig sequence of the reference genome, while the other end fully or partially maps to an unexpected locus. The designation “singleton” refers to one end of a read pair that does not map to the reference genome, whereas “unmapped read pairs” refers to both ends of a read pair that do not map to the reference genome (Fig. 1). Discordant read pairs may provide insights regarding LTR-related loci as the anchor^37^. RetroSeq software^37^ was used to detect non-reference transposon elements (TEs) using mismatched reads. The process flow is shown in Fig. 2. The LTR sequences were based on PERV-LTR sequences acquired from the National Center for Biotechnology Information (NCBI, Bethesda, MD, USA) under accession Nos. AF435966, AF546883-AF546887, AJ279057, AJ298073-AJ298075, AY312534-AY312550, EF133960, EU789636, and HQ540595. The reference genome was Sscrofa11.1. which contains only chromosomes 1–18 and X. In the RetroSeq “call” step, the TE insertion loci (breakpoints) were inferred using reads detected during the “discover” phase, as previously reported^38^. The “call” step read option was set to ≥ 10 to reduce false positives. The maximum read depth option per call was set to 10,000 to increase BAM coverage. All other RetroSeq options were used at their default values. At least seven filter level breakpoints were employed. Calls within 500-bp of a detected breakpoint were considered identical and were excluded. The IGV^39^ was used to detect loci containing TSDs 4–5 bp long. The loci were presumed to be TSD if they mapped on reads detected during the “discover” phase either from the 5′ or 3′ side, overlapping by 4–5 bp (Fig. 2). The 5′ and 3′ reads mapping within 150 bp of the TSD were extracted with SAMtools^40^ (Fig. 2). The read sets were used to generate contig sequences by local de novo assembly with CAP3 software^41^. The presence of the LTR sequence was confirmed from the contig sequence created by CAP3.

### PCR amplification and nucleotide sequencing determination of non-reference PERVs

For the loci wherein non-reference LTRs were identified, PCR was performed to confirm the presence of PERVs. The final PCR mixture consisted of 0.4 U KOD FX neo Taq (TaKaRa Bio Inc., Kusatsu, Shiga, Japan), 10 μL of 2 × KOD FX neo buffer (TaKaRa Bio Inc.), 1.6 μL of deoxynucleotide triphosphate (dNTP; 2.5 mL of each type; TaKaRa Bio Inc.), 1.2 μL of 10 µM forward and reverse primers per site, and 10 ng DNA in a total volume of 20 μL. The primers used for PCR are listed in Suppl. Table S1. The PCR program comprised a denaturation step at 95 °C for 2 min, followed by 40 cycles of 95 °C for 10 s and 68 °C for 10 min. After PCR, the 8000–10,000-bp DNA band was purified and subjected to a second PCR using the primers for the PERV *pol* region^6^. The amplicon of the second PCR cycle was sequenced with an ABI3130 BigDye Terminator v. 3.1 Cycle Sequencing Kit (Applied Biosystems, Foster City, CA, USA) and analyzed with a ABI3130 Genetic Analyzer (Applied Biosystems) to detect the presence of PERV sequences. The types of PERVs were determined using the Basic Local Alignment Search Tool^42^ according to the partial sequences of the PERV *pol* region.

### LTR structure and phylogenetic tree analysis

The detected non-reference LTRs were aligned with Multiple Sequence Comparison by the Log-Expectation program^43,44^. The phylogenetic tree was inferred by the maximum likelihood method and the kimura three-parameter model included in Molecular Evolutionary Genetics Analysis software (MEGA X) to classify the non-reference LTRs^45–47^.

### qRT-PCR for copy number estimation

The numbers of PERV *pol* gene copies in the three pigs were estimated by qRT-PCR according to a previously reported method. *β-actin* (*ACTB*) was used as an endogenous control (reference) gene. The primers and probes were the same as those used in a previous study^6^. The PCR amplicons of these primer sets were cloned into the pCR-TOPO2.1 vector. The standard curves for absolute quantification were plotted using serial dilutions of linearized DNA from *ACTB* and *pol* gene plasmid clones. The qRT-PCR mixtures used for DNA amplification were prepared by adding 7.5 µL of 2 × TaqMan Gene Expression Master Mix (Applied Biosystems), 10 µM of each forward and reverse primer, 5 µM of each of the *ACTB* and *pol* gene probes, and 5 ng DNA from each pig. Distilled water was added to make up a final volume of 15 µL. The PCR program consisted of a denaturation step at 95 °C for 2 min, followed by 40 cycles of 95 °C for 15 s and 60 °C for 1 min. A dissociation curve was plotted to confirm the specificity of the amplified products. *ACTB* and *pol* were quantified using standard curves plotted with plasmid DNA. The PERV gene copy numbers were estimated as previously described^6^.

## Supplementary Information

Supplementary Information 1.

Supplementary Information 2.

Supplementary Information 3.

Supplementary Information 4.

Supplementary Information 5.

## Data Availability

Raw data of whole-genome sequencing are available at the DDBJ sequence read archive (DRA) under Accession No. DRA013149.
